# A molecular determinant of phosphoinositide affinity in mammalian TRPV channels

**DOI:** 10.1038/srep27652

**Published:** 2016-06-13

**Authors:** Phanindra Velisetty, Istvan Borbiro, Marina A. Kasimova, Luyu Liu, Doreen Badheka, Vincenzo Carnevale, Tibor Rohacs

**Affiliations:** 1 Department of Pharmacology, Physiology and Neuroscience, Rutgers – New Jersey Medical School, Newark, NJ 07103, USA; 2 Institute for Computational Molecular Science at Temple University in Philadelphia, PA, 19122, USA

## Abstract

Phosphatidylinositol 4,5-bisphosphate [PI(4,5)P_2_] is an important cofactor for ion channels. Affinity for this lipid is a major determinant of channel inhibition by depletion of PI(4,5)P_2_ upon phospholipase C (PLC) activation. Little is known about what determines PI(4,5)P_2_ affinity in mammalian ion channels. Here we report that two members of the Transient Receptor Potential Vanilloid (TRPV) ion channel family, TRPV5 and TRPV6 lack a positively charged residue in the TM4-TM5 loop that was shown to interact with PI(4,5)P_2_ in TRPV1, which shows high affinity for this lipid. When this positively charged residue was introduced to either TRPV6 or TRPV5, they displayed markedly higher affinities for PI(4,5)P_2_, and were largely resistant to inhibition by PI(4,5)P_2_ depletion. Furthermore, Ca^2+^-induced inactivation of TRPV6 was essentially eliminated in the G488R mutant, showing the importance of PLC-mediated PI(4,5)P_2_ depletion in this process. Computational modeling shows that the introduced positive charge interacts with PI(4,5)P_2_ in TRPV6.

Transient Receptor Potential Vanilloid 6 (TRPV6) is a constitutively active, Ca^2+^ selective, inwardly rectifying ion channel. It constitutes the initial entry step in the transcellular Ca^2+^ transport process in intestinal epithelial cells^1^. Its genetic deletion in mice causes disturbances in extracellular Ca^2+^ homeostasis^2^,^3^. Ca^2+^ influx via TRPV6 in the epidydimis is required for male fertility in mice^4^. In the absence of divalent cations, TRPV6 also conducts monovalent currents, which are much larger than Ca^2+^ currents and thus are widely used to assess the activity of these channels. The activity of TRPV6, similarly to most TRP channels, depends on phosphatidylinositol 4,5-bisphosphate [PI(4,5)P_2_]^5^,^6^.

TRP channels are activated by diverse cellular and environmental signals^7^. Their only known common functional feature is modulation by phosphoinositides, mainly PI(4,5)P_2_. The vast majority of TRP channels are positively regulated by PI(4,5)P_2_, but negative effects may coexist on some TRP channels, such as TRPV1 or some TRPC-s see recent reviews for further details^8^,^9^,^10^. PI(4,5)P_2_ and its precursor PI(4)P are the most abundant phosphoinositides in the plasma membrane. While PI(4,5)P_2_ received the most attention, PI(4)P may also play distinct roles in regulation of some TRP channels^11^,^12^. Ion channels, including TRPs, are generally thought to be activated by phosphoinositides via direct interactions between the negatively charged head group of the lipid and (mainly) positively charged residues in the cytoplasmic domains of the channel protein. How TRP channels interact with this phospholipid has been the subject of intensive research and many different cytoplasmic regions have been implicated. Most efforts focused on the roles of various parts of the cytoplasmic C-terminus^13^,^14^,^15^,^16^.

The apparent affinity of an ion channel to phosphoinositides is a major determinant of its inhibition by PI(4,5)P_2_ depletion upon activation of phospholipase C (PLC)^10^,^17^,^18^. It is not very well understood, however, what determines the affinity of mammalian ion channels for PI(4,5)P_2_. Earlier work on mammalian Kir channels found that PI(4,5)P_2_ interacting residues are generally conserved between family members, and the difference between high and low affinity channels could be traced to a subtle conformational change caused by a relatively conservative substitution of Leucin to Isoleucin^19^. Recently it was shown that a Kir channel from a marine sponge *Amphimedon queenslandica* displays very weak interactions with PI(4,5)P_2_, and lacks two key positively charged PI(4,5)P_2_ interacting residues that are highly conserved in mammalian Kir channels^20^. Reintroduction of the two positively charged residues substantially strengthened the interactions of the invertebrate Kir channel with PI(4,5)P_2_^20^.

Here we report that two members of the Transient Receptor Potential Vanilloid (TRPV) ion channel family, TRPV5 and TRPV6, lack a positively charged residue in the TM4-TM5 loop, equivalent of which was shown to interact with PI(4,5)P_2_ in TRPV1^21^, a channel with high affinity for phosphoinositides^22^. When we reintroduced this positive charge to TRPV6 (G488R), the mutant showed markedly slower rundown in excised patches and increased apparent affinity for dioctanoyl (diC_8_) PI(4,5)P_2_. The mutant channel was completely resistant to depletion of PI(4,5)P_2_ by the voltage-sensitive PI(4,5)P_2_ 5′-phosphatase from *Ciona intestinalis* (ciVSP)^23^, and showed essentially no Ca^2+^-induced inactivation, presumably because of its increased affinity for PI(4,5)P_2_. Computational modeling showed that in the mutant TRPV6 the introduced 488R residue interacted with PI(4,5)P_2_.

## Results

**Figure 1:**
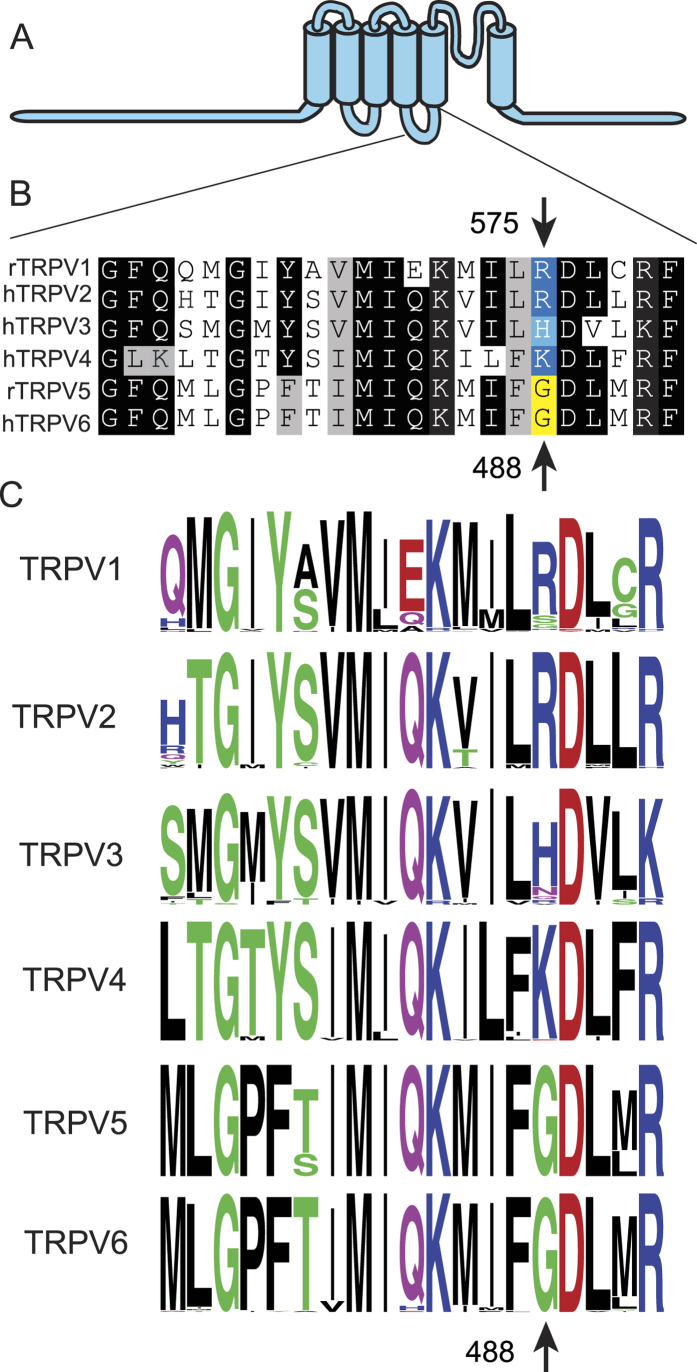
Sequence alignment of TRPV channels in the TM4-TM5 loop. (**A**) Cartoon of TRPV channels (**B**) Multiple sequence alignment of the TM4-TM5 loop of selected mammalian TRPV channels (**C**), Sequence logos for the 6 different TRPV channels based on a total of 447 sequences previously collected and aligned^57^.

**Figure 2:**
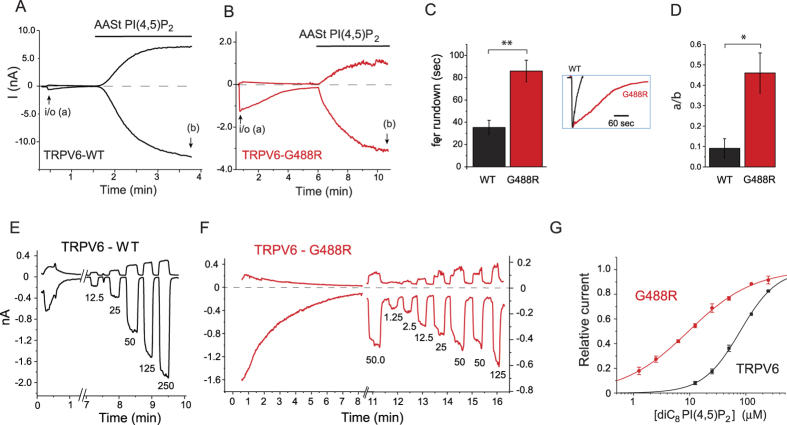
Increased apparent affinity of TRPV6-G488R for PI(4,5)P_2_. Excised inside-out patch clamp recordings on *Xenopus* oocytes expressing either wild-type or the G488R mutant of TRPV6 were performed as described in the Materials and Methods section. (**A**,**B**) representative traces, currents at −100 and 100 mV are plotted. The establishment of the inside-out (i/o) configuration is indicated by the arrows, the application of 10 μM AASt PI(4,5)P_2_ is shown by the horizontal lines. In both measurements the patch formed a vesicle after excision, explaining the very low currents at the beginning of the trace. The vesicle was disrupted by a short exposure to an air bubble, establishing the inside-out configuration. (**C**) Summary of the time constants for rundown of the wild-type and the G488R mutant, inset shows the two representative traces normalized to the peak current levels either after excision, or vesicle breakage. (**D**) Summary of current levels right after excision, or vesicle breakage (a) divided by current levels at the end of AASt PI(4,5)P_2_ application (b) (n = 13 for wild-type and n = 8 for G488R). (**E**–**G**) DiC_8_ PI(4,5)P_2_ dose response measurements in TRPV6 and TRPV6-G488R. Excised inside-out patch clamp recordings on *Xenopus* oocytes expressing either wild-type or G488R TRPV6 were performed as described in the Materials and Methods section. (**E**,**F**) representative traces, currents at −100 and 100 mV are plotted. Numbers denote the concentration (μM) for applications of diC_8_ PI(4,5)P_2_. (**G**) Summary for the fits with the Hill equation (n = 3–8 for each concentration), data were normalized to the maximum effect obtained from the Hill fits, see methods for further details. The EC_50_ for the wild-type channel was 78.9 ± 6.5 μM, with a Hill coefficient of 1.32 ± 0.05. The EC_50_ for the G488R mutant was 9.46 ± 0.61 μM, with a Hill coefficient of 0.75 ± 0.02.

We have inspected the multiple sequence alignment of TRPV channels, and noticed that the equivalents of residue R575 in the TM4-TM5 loop that was shown to interact with PI(4,5)P_2_ in TRPV1^21^ is a G in TRPV5 and TRPV6, whereas it is a positively charged residue in all other TRPV channels (Fig. 1). We hypothesized that the lack of this positive charge is responsible for the low apparent affinity of TRPV6 for PI(4,5)P_2_. We have mutated this residue to an Arginine in TRPV6 (G488R), and performed a number of experiments to evaluate the apparent affinity of the mutant and the wild-type channels for PI(4,5)P_2_. Figure 2 shows an experiment in the excised inside-out configuration on TRPV6 channels expressed in *Xenopus* oocytes. Activity of the wild-type channels showed fast and marked rundown after excision, similar to many PI(4,5)P_2_-sensitive ion channels. Rundown is generally accepted to be caused by dephosphorylation of PI(4,5)P_2_ in the excised patches by lipid phosphatases, and its velocity was shown to depend on the apparent affinity of the channel for PI(4,5)P_2_, i.e. channels with low affinity run down fast, whereas high affinity channels run down slowly^19^,^24^. The time constant of rundown for the wild-type TRPV6 was markedly faster than the G488R channel (Fig. 2C), indicating that the mutant had higher affinity for PI(4,5)P_2_.

Next we applied phosphoinositides to the inner surface of the excised patch, to directly assess the effects of the lipids. First we used the natural long acyl chain Arachidonyl-stearyl (AASt) PI(4,5)P_2_, which is the most abundant form in the plasma membrane. This lipid forms micelles in aqueous solutions, and activates ion channels by incorporation of the lipid vesicle into the patch membrane. The effect of this lipid on ion channels usually develops slowly and it is essentially irreversible^25^. The wild-type channel showed ~10 times higher current levels upon the application of 10 μM AASt PI(4,5)P_2_ compared to current levels after excision (Fig. 2A,D). This shows that endogenous PI(4,5)P_2_ levels only induce a fraction of maximal activity, which is consistent with low affinity for PI(4,5)P_2_^26^. The G488R mutant on the other hand showed only a ~2 fold increase compared to current levels after excision (Fig. 2B,D). This again is consistent with increased apparent affinity of the mutant for PI(4,5)P_2_.

An often-used method to have a more direct estimate of the apparent affinity of the channel for PI(4,5)P_2_ is performing dose-response measurements with short acyl chain diC_8_ PI(4,5)P_2_^27^. These synthetic lipids are efficient tools to study ion channels, because they are water-soluble, thus their monomer concentrations are high, and they incorporate into the patch membrane quickly. Their effects are also reversible, because they readily diffuse out of the membrane upon cessation of the stimulus^25^. Figure 2E–G shows that the activation curve for diC_8_ PI(4,5)P_2_ is shifted to the left in the G488R mutant, compared to the wild-type TRPV6, again consistent with increased affinity for PI(4,5)P_2_. The EC_50_ values for the wild-type TRPV6 and G488R mutant were 78.9 and 9.46 μM respectively (Fig. 2G).

Ion channels with low apparent affinities for PI(4,5)P_2_ are inhibited by depletion of PI(4,5)P_2_ more than channels with higher affinities^19^. Therefore we tested inhibition of the wild-type TRPV6 and the G488R mutant by the voltage inducible 5′-phosphatase ciVSP, which depletes PI(4,5)P_2_ by converting it to PI(4)P^23^. To perform these measurements, we coexpressed TRPV6 channels with ciVSP in HEK cells, because in our hands, the ciVSP construct is toxic to oocytes, and induces large leak currents. Monovalent currents via TRPV6 were induced by removing extracellular Mg^2+^, and chelating trace amounts of Ca^2+^ with EGTA at a holding potential of −60 mV. We measured monovalent currents because they are larger and more readily detectable than Ca^2+^ currents and they are widely used to assess TRPV6 activity^1^,^5^,^28^. Also this way whole-cell patch clamp data are more comparable to oocytes, where we also measured monovalent currents, because the endogenous Ca^2+^-induced Cl^−^ currents would mask Ca^2+^ currents.

**Figure 3:**
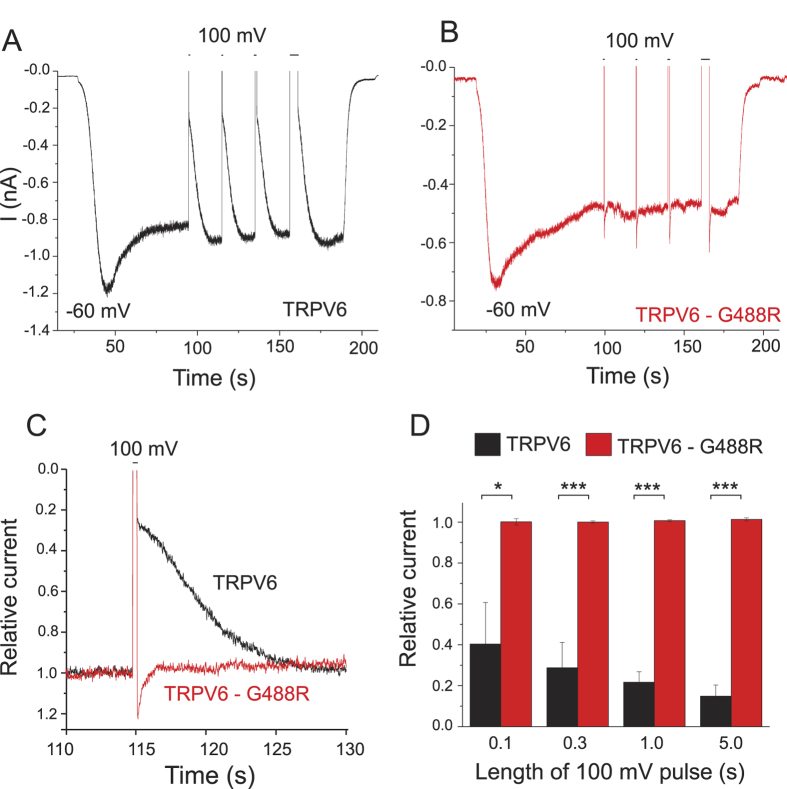
Inhibition of TRPV6 but not TRPV6-G488R by ciVSP. Whole-cell patch clamp experiments were performed on HEK cells transfected with ciVSP, and either the wild-type TRPV6, or the G488R mutant, as described in the Materials and Methods section. At the beginning of the experiment, monovalent currents were initiated with the application of a solution devoid of Mg^2+^ and Ca^2+^ and supplemented with 1 mM EGTA. At the end of the experiment, monovalent currents were terminated by a solution devoid of EGTA and containing 1 mM Mg^2+^. The holding potential was −60 mV, and successive depolarizations to 100 mV with increasing durations were applied to activate ciVSP. (**A**,**B**) representative measurements on full time scales. (**C**) Representative traces on an expanded time scale, with currents normalized to levels before the 0.3 s depolarizing pulse to 100 mV. (**D**) Summary, current amplitudes were normalized to levels before each depolarization, and values immediately after the depolarizing pulses were plotted for wild-type TRPV6. For G488R the transient increases in current after returning to −60 mV were excluded from the analysis (n = 4–5).

As shown in Fig. 3., when a depolarizing pulse as short as 0.1 s to 100 mV was applied, in cells expressing ciVSP and wild-type TRPV6, monovalent currents at −60 mV were inhibited by ~60%. Recovery was full within ~10 s consistent with earlier reports on other channels, such as TRPM8^29^ or voltage gated Kv7 (KCNQ) channels^30^, reflecting fast resynthesis of PI(4,5)P_2_ by phosphatidylinositol 4-phosphate 5′-kinases (PIP5K). Longer depolarizing pulses induced larger inhibitions, which reached ~85% at 5 s. When the G488R mutant was coexpressed with ciVSP, however, the depolarizing pulses did not evoke any inhibition even when they lasted for 5 s (Fig. 3B,D). The inactive ciVSP mutant C363S^23^,^29^ did not show any inhibition either on the wild-type TRPV6 (Fig. S1A,B,F), or on the G488R mutant (Fig. S1C,D,F). To test the importance of a positive charge at this location, we also mutated the G488 residue to Lysine; this G488K mutant was not inhibited by depolarizing pulses in cells co-expressing ciVSP (Fig. S1E,F).

**Figure 4:**
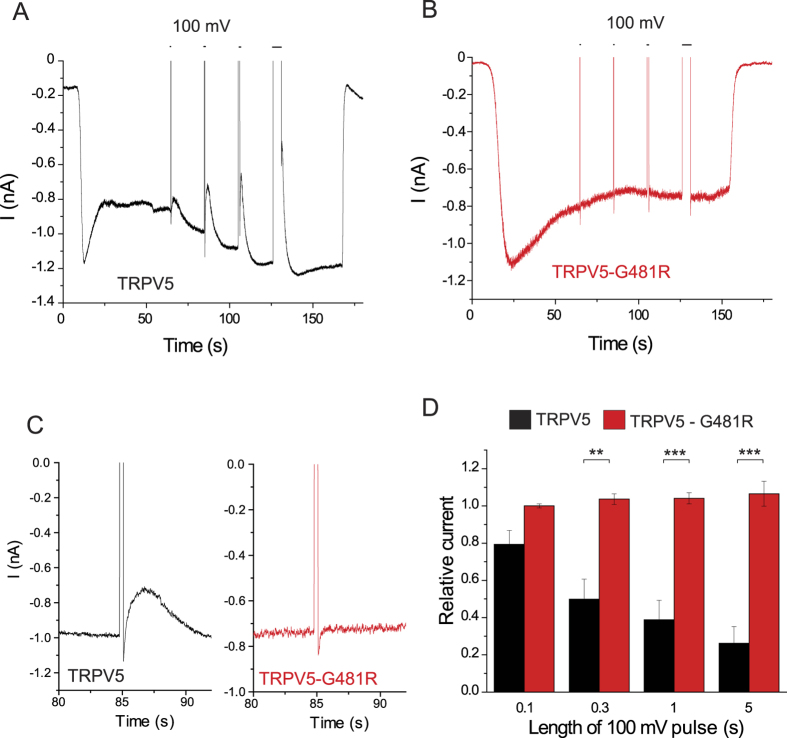
Inhibition of TRPV5 but not TRPV5-G481R by ciVSP. Whole-cell patch clamp experiments were performed on HEK cells transfected with ciVSP and either the wild-type TRPV5 or the G481R mutant, as described in the Materials and Methods section. At the beginning of the experiment, monovalent currents were initiated with the application of a solution devoid of Mg^2+^ and Ca^2+^ and supplemented with 1 mM EGTA. At the end of the experiment, monovalent currents were terminated by a solution devoid of EGTA and containing 1 mM Mg^2+^. The holding potential was −60 mV, and successive depolarizations to 100 mV with increasing durations were applied to activate ciVSP. (**A**,**B**) representative measurements, (**C**), Traces show the 0.3 s long depolarizing pulse from same measurements on an expanded time scale. (**D**) Summary, current levels were normalized to those measured before each depolarization, and values after the depolarizing pulses (excluding the brief transient currents) were plotted for wild-type TRPV5 and for G481R (n = 4–5).

We also generated the mutation G481R in TRPV5, which is equivalent to G488R in TRPV6. Figure 4A shows that activation of ciVSP inhibited the wild-type TRPV5 channel, but somewhat longer depolarizing stimuli were needed to inhibit this channel compared to TRPV6, suggesting higher apparent affinity. The G481R mutant of TRPV5 was not inhibited even by the longest depolarizing pulses (Fig. 4B,C), which is consistent with high affinity for PI(4,5)P_2_. In 3 out of 5 cells, including the representative trace in Fig. 4A, wild-type, but not mutant TRPV5 current amplitudes showed a small increase after recovery from inhibition. We have not investigated this phenomenon further.

Most cells expressing ciVSP and TRPV6-G488R or TRPV5-G481R displayed a transient “tail-current” after returning from +100 mV to −60 mV, which were distinct from capacitative currents (Figs 3B,C and 4B,C). Similar current was also seen in one cell expressing wild-type TRPV5 (Fig. 4A,C), but in wild-type TRPV6 expressing cells they were either absent or masked by the rapidly developing TRPV6 current inhibition (Fig. 3A). Gating currents of ciVSP^31^ may have contributed to these “tail currents”, but they were not seen consistently in every cell, see examples in Fig. S1. We have not further investigated the nature of these transient currents.

**Figure 5:**
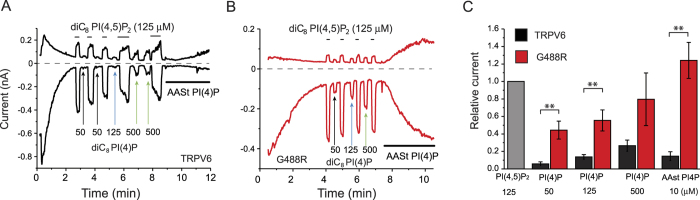
Increased activation of TRPV6-G488R by PI(4)P. Excised inside-out patch clamp recordings on *Xenopus* oocytes expressing either wild-type or G488R TRPV6 were performed as described in the Materials and Methods section. (**A**,**B**) representative traces, currents at −100 and 100 mV are plotted. Arrows show application of various concentrations of diC_8_ PI(4)P_2_ (μM). In between applying different concentrations of diC_8_ PI(4)P, 125 μM diC_8_ PI(4,5)P_2_ was applied as a normalizing stimulus. At the end of each experiment the patch was perfused with 10 μM AASt PI(4)P. (**C**) Summary of the data normalized to the current levels evoked by 125 μM diC_8_ PI(4,5)P_2_ in each patch (n = 4). The p value (p = 0.066) for the difference between the effects of 500 μM diC_8_ PI(4)P on TRPV6 and the G488R mutant did not reach the cutoff for statistical significance.

CiVSP converts PI(4,5)P_2_ into PI(4)P, thus, if the latter also activates a channel, we expect either no, or less inhibition depending on the extent of activation by PI(4)P^12^,^15^. To assess the possibility that the lack of inhibition of the mutant channel by ciVSP was not only caused by increased affinity, but also altered phosphoinositide specificity, we tested if TRPV6-G488R is activated by PI(4)P. Wild-type TRPV6 channels were essentially not activated by diC_8_ PI(4)P, only at very high concentrations (Fig. 5A,C), which is consistent with earlier results^5^, and with the robust inhibition by ciVSP. AASt PI(4)P (10 μM) also only induced a negligible activation of the wild-type TRPV6, which was less than 5% of the current levels after excision (Fig. 5A,C). The G488R mutant on the other hand showed marked activation both by diC_8_ PI(4)P and by 10 μM AASt PI(4)P (Fig. 5B,C), but the activation by PI(4)P was less than that induced by PI(4,5)P_2_ for both the short and long chain isoforms. Current amplitudes induced by AASt PI(4)P on average were 49.2 ± 12.5% (n = 4) of that seen right after patch excision, while AASt PI(4,5)P_2_ induced a more than 2-fold increase in the G488R mutant (Fig. 2B,D).

**Figure 6:**
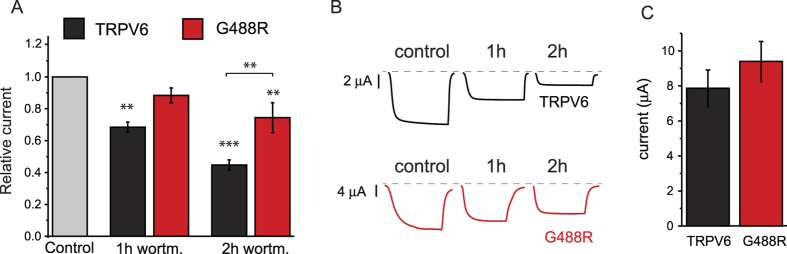
Altered inhibition of TRPV6-G488R by wortmannin. Two electrode voltage clamp recordings on *Xenopus* oocytes expressing either wild-type or G488R TRPV6 were performed as described in the Materials and Methods section. Current levels were measured on individual oocytes expressing either TRPV6 or the G488R mutant. The oocytes were treated with 35 μM wortmannin to inhibit PI4K then currents were measured at 1 hour, then after continued treatment with wortmannin, currents were measured again at 2 h on the same oocytes. (**A**) shows summary data normalized to current levels before wortmannin treatment in the same oocyte for each measurement. The difference between the wild-type and the mutant at 1 h wortmannin treatment did not reach statistical significance (p = 0.112). Asterisks above individual bars show significant difference from currents before wortmannin treatment. (**B**) individual representative traces. (**C**) absolute current levels before wortmannin treatment (n = 11–13).

To use an alternative way to deplete phosphoinositides, we treated oocytes with high concentrations of wortmannin to inhibit phosphatidylinositol 4-kinases (PI4K). Wortmannin at low nanomolar concentrations is specific for phosphoinositide 3-kinases (PI3K), but at high micromolar concentrations it also inhibits PI4K^32^. We found earlier that 35 nM wortmannin did not inhibit TRPV6 channels^33^. Here we incubated oocytes expressing either wild-type TRPV6 or the G448R mutant with 35 μM wortmannin for 1 and 2 hours. Basal current amplitudes were measured in untreated oocytes, then the same individual oocytes were incubated with 35 μM wortmannin and currents were measured at 1 h, then again after 2 h incubation. Current amplitudes in Fig. 6A were normalized to the current before wortmannin treatment for each oocyte. Wortmannin inhibited the wild-type channels more than the G488R mutant, and this difference was statistically significant at 2 h. Basal current amplitudes were not different between wild-type and mutant channels (Fig. 6C).

**Figure 7:**
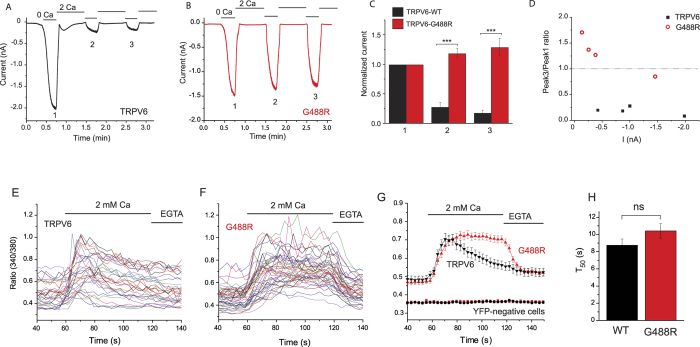
Altered Ca^2+^-induced inactivation in the G488R mutant. Whole-cell patch clamp experiments were performed on HEK cells transfected with either the wild-type TRPV6 or the G488R mutant, as described in the Materials and Methods section. The holding potential was −60 mV. (**A**,**B**) Representative traces, monovalent currents were initiated with the application of a solution devoid of Mg^2+^ and Ca^2+^ and supplemented with 1 mM EGTA, and a solution containing 2 mM Ca^2+^ was applied as indicated by the horizontal lines to evoke Ca^2+^-induced inactivation. (**C**) Summary of the data, normalized to the peak amplitudes of the first monovalent current at the time points indicated by the numbers (n = 4). (**D**) Individual experiments are plotted with current levels on the x-axis and inactivation levels on the y-axis plotted as the ratio between the current levels at the 3^rd^ and the 1^st^ application of 0 Ca^2+^ solution. (**E**–**G**) Ca^2+^ influx is transient in the wild-type TRPV6, but sustained in the G488R mutant. Ca^2+^ imaging experiments in cells expressing the wild-type TRPV6 or the G488R mutant channels were performed as described in the methods section on cells loaded with the low affinity Ca^2+^-sensitive dye Fura-2-lowAff. At the beginning of the experiment the cells were kept in nominally Ca^2+^ free medium, for at least 30 minutes, then Ca^2+^ influx was initiated by increasing Ca^2+^ to 2 mM. At the end of the experiment Ca^2+^ influx was terminated by a Ca^2+^ free solution containing 2 mM EGTA. (**E**,**F**) individual traces in cells expressing TRPV6 or G488R, (**G**) summary, showing mean ± SEM (n = 31 for TRPV6 and n = 44 for G488R). Bottom traces show responses of cells without the transfection marker YFP from the same cover slips. (**H**) time required to reach 50% of the maximal increase upon Ca^2+^ application is plotted for cells transfected with wild-type (n = 31) and G488R TRPV6 (n = 44) (p = 0.13).

Depletion of PI(4,5)P_2_ by activation of a Ca^2+^-sensitive PLC isoform has been shown to play a role in Ca^2+^-induced inactivation of TRPV6^5^,^34^. The G488 mutant has high affinity for PI(4,5)P_2_ and it is not inhibited upon PI(4,5)P_2_ depletion by ciVSP (Fig. 3). Thus we tested if the mutant shows altered Ca^2+^-induced inactivation, as would be expected if PI(4,5)P_2_ depletion plays a role in this phenomenon. We used a protocol described by us earlier^5^, where monovalent TRPV6 current measurements are interspersed by applications of 2 mM Ca^2+^ (Fig. 7A). Upon application of 2 mM Ca^2+^, monovalent currents are rapidly inhibited due to direct Ca^2+^ block. The decrease of the amplitudes of the second and third induction of monovalent currents reflects Ca^2+^-induced inactivation, due to the combined effect of PI(4,5)P_2_ depletion and CaM^33^. We use the decrease in monovalent current amplitudes to assess Ca^2+^-induced inactivation, because the Ca^2+^ currents, detectable after applying 2 mM Ca^2+^, are quite variable, and often seemingly absent in non-inactivating mutants because they are difficult to differentiate from leak and/or background currents^33^. In contrast to the wild-type TRPV6 channel, the G488R mutant showed no Ca^2+^-induced inactivation in this protocol (Fig. 7B). The G488R currents were smaller on average in these experiments, but even at similar current amplitudes the difference between Ca^2+^-induced inactivation of the two channels was marked (Fig. 7D).

When we pooled the monovalent current amplitudes measured in HEK cells at −60 mV from experiments in Figs 3 and 7 and Fig. S1, on average, wild-type TRPV6 had −1.15 ± 0.19 nA currents (n = 14), whereas the G488R mutant −0.66 ± 0.12 nA (n = 16, p = 0.038). In contrast, in oocytes current amplitudes were similar, with a small trend for higher amplitudes in the mutant (Fig. 6C). We also compared monovalent currents in wild-type and mutant TRPV6 channels using a voltage step protocol (Fig. S2), where the mutant and wild-type channels showed similar inward rectification. The mutant had somewhat smaller amplitudes, but the difference was not statistically significant. The Supplemental Table summarizes current amplitudes in the wild-type and mutant channels from the different measurements. From the higher affinity of the mutant for PI(4,5)P_2_, and the finding that resting PI(4,5)P_2_ levels do not saturate the wild-type channel, it would ensue that the G488R mutant has higher current amplitudes. It has to be noted however, that the mutant showed essentially no Ca^2+^-induced inactivation, thus it is likely to overload cells with Ca^2+^. This could damage the cells with high expression levels of this channel, thus excluding them from our measurements and alter conclusions. Constitutive Ca^2+^ influx could also induce mechanisms to down regulate channel activity, thus overall it is difficult to draw firm conclusions from these current amplitude measurements.

We also measured the kinetics of Ca^2+^ signals evoked by influx through the wild-type and mutant TRPV6 channels by Ca^2+^ imaging (Fig. 7E–H). We used a low affinity dye (Fura-2-LowAff), to avoid saturation of the dye upon massive Ca^2+^ influx, which could lead to underestimating the difference between two large Ca^2+^ signals^12^. Cell were kept in nominally Ca^2+^ free medium for at least 30 min before the measurements, and Ca^2+^ influx was initiated by increasing extracellular Ca^2+^ to 2 mM. Cells expressing the wild-type channel showed a transient increase in cytoplasmic Ca^2+^ (Fig. 7E,G), which is consistent with earlier results^5^,^35^, and reflects Ca^2+^-induced inactivation of the channel. In cells expressing the G488R mutant, Ca^2+^ signals using the same protocol were sustained (Fig. 7F,G), which is consistent with the impaired Ca^2+^-induced inactivation in our patch clamp measurements. The peak amplitude of the Ca^2+^ signal was similar between wild-type and mutant channels, with the mutant being slightly larger, which is consistent with the average current amplitudes being comparable. Cells on the same cover slips that did not have YFP fluorescence (transfection marker), did not show any increase in cytoplasmic Ca^2+^ (Fig. 7G, lower traces), showing that the Ca^2+^ increase was due to influx through the channel. We also calculated the initial rate of rise of Ca^2+^, which may be a better indication of the initial Ca^2+^ influx than the extent of increase in cytoplasmic Ca^2+^. The mutant showed a somewhat less steep increase, but this difference was not statistically significant. We quantified this by measuring the time required to reach half of the peak amplitude, and the data are plotted in Fig. 7H.

**Figure 8:**
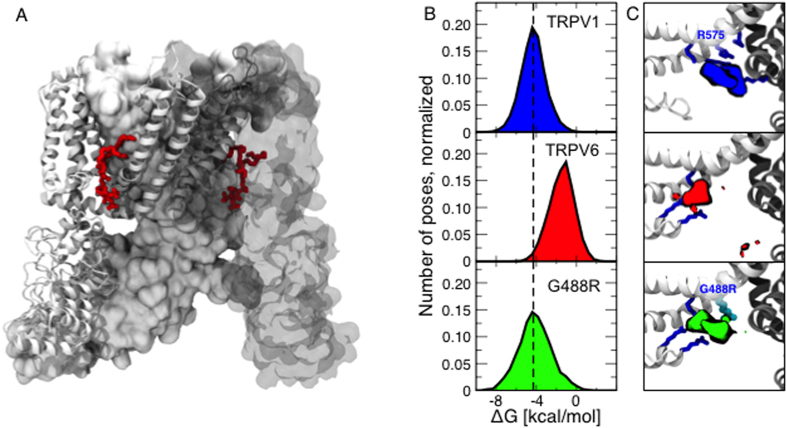
Docking of PI(4,5)P_2_ to the G488R mutant and wild-type TRPV6 and to TRPV1. (**A**) PI(4,5)P_2_ (red) docked to the homology model of TRPV6 based on the structure of TRPV1. (**B**,**C**) Binding of PI(4,5)P_2_ to TRPV1 (top row), TRPV6 (second row from top), TRPV6-G488R (third row). (**B**) Distributions of binding free energies. (**C**) Binding site location highlighted by phosphate atoms density and coordinating positively charged residues (sticks).

In order to estimate the PI(4,5P)_2_ binding free energy of the wild-type TRPV6 and the G488R mutant we performed a series of docking simulations^36^. Since no atomic resolution structure of TRPV6 has been determined yet, we generated a theoretical model using the comparative homology modeling approach^37^ with the structure of TRPV1 (pdb code 3J5P) as a template (Fig. 8A). This modeling approach produces a set of distinct structures compatible with the template; we grouped these structures (using a clustering procedure) and prioritized them according to the ROSETTA empirical score. To ascertain that the predicted PI(4,5)P_2_-TRPV6 binding mode does not depend crucially on the choice of the representative structure, we repeated the docking simulations using the 40 models of TRPV6 with the largest score. For comparison, we performed the same docking simulations also for TRPV1.

In the case of TRPV1, the average free energy of the accepted poses (see Methods) is −4.4 kcal/mol, which corresponds to a relatively high affinity (Fig. 8B, first row). The density of the PI(4,5)P_2_ phosphorus atoms in the binding site is shown in Fig. 8C, first row: the lipid is in interaction with R575 corresponding to G488 in TRPV6 (see Fig. 1). The fact that these findings are in perfect agreement with the results of a recent independent investigation^21^, supports the notion that our computational protocol is robust and gives us confidence in the predictions about TRPV6.

In the case of TRPV6, for the wild-type, the average free energy of the accepted poses is −1.2 kcal/mol (Fig. 8B, second row). In agreement with the experimental data, our findings indicate that TRPV6 has lower PI(4,5)P_2_ affinity compared to TRPV1. However, for the G488R mutant, the average free energy of the accepted poses is shifted toward negative values and corresponds to −4.4 kcal/mol (Fig. 8B, third row), which indicates that the mutant has higher affinity for PI(4,5)P_2_ compared to the wild-type, in line with the experimental results presented in this work. The higher affinity of the G488R mutant is due to the fact that the mutated G488R residue establishes additional favorable interactions with the PI(4,5)P_2_ moiety (Fig. 8C, second and third rows for the wild-type and the mutant respectively).

## Discussion

PI(4,5)P_2_ is required for the activity of most TRP channels, but how this lipid opens them is unclear. The conserved nature of the positive effect of PI(4,5)P_2_ presumes a relatively conserved mechanism and binding site. Yet, the picture of which parts of the channel interact with PI(4,5)P_2_ is confusing^8^,^9^,^38^. Most experimental work focused on various parts of the cytoplasmic C-terminus^13^,^14^,^15^,^16^,^39^, and some work suggests involvement of the cytoplasmic N-terminus^40^. PI(4,5)P_2_ is a membrane phospholipid, thus a physiological PI(4,5)P_2_ binding site has to be located close to the plasma membrane. Before high-resolution nearly full-length structures were available, membrane proximity of a cytoplasmic region was difficult to assess. The relatively recent side-chain resolution structure of TRPV1^41^,^42^ makes it possible to build reliable homology models of TRPV channels and computationally dock PI(4,5)P_2_.

A recent article proposed that the R575 residue in the TM4-TM5 loop of TRPV1 serves as part of a PI(4,5)P_2_ binding site by using computational modeling, and showing that the diC_8_ PI(4,5)P_2_ dose-response of the R575Q mutant was shifted to the right^21^. This region is analogous to the proximal N-terminal cytoplasmic domain of the two TM Kir channels. Based on earlier mutagenesis experiments^43^ and the co-crystal structures of PI(4,5)P_2_ with two different Kir channels^44^,^45^, positively charged residues in this this region, together with residues in the proximal C-terminus, interact with PI(4,5)P_2_. This analogy with Kir channels also makes the TM4-TM5 loop an attractive candidate for playing roles in PI(4,5)P_2_ activation in TRPV channels.

Regulation of TRPV1 however is complex. The channel needs PI(4,5)P_2_ for activity, but the lipid alone does not activate it at physiological membrane potentials; opening requires an additional stimulus, such as heat, capsaicin, or low pH. TRPV1 is also weakly voltage dependent^46^, and extreme depolarizations can open it together with PI(4,5)P_2_^21^. Both capsaicin and heat shift the voltage sensitivity of the channel to more negative voltages^46^. Capsaicin also shifts the diC_8_ PI(4,5)P_2_ dose response to lower concentrations^47^. Furthermore the dose response relationships for both PI(4,5)P_2_ and PI(4)P are shifted to the left at highly positive voltages^47^. Heat, capsaicin, voltage and PI(4,5)P_2_ thus regulate the channel in a complex fashion, each factor influencing the others. Given this complexity, it is difficult to distinguish between primary and secondary effects of a mutation. The R575Q mutation for example not only shifted the PI(4,5)P_2_ dose response, but also affected the voltage activation of TRPV1^21^. Similarly complex interplay between voltage, cold, menthol and PI(4,5)P_2_ was described for TRPM8 channels^14^,^46^.

TRPV6 is constitutively active, and it is neither voltage dependent, nor it requires any other stimulus than PI(4,5)P_2_ to open. Thus we argue, that mutation phenotypes in this channel are more straightforward to interpret than in other, more complex TRP channels. TRPV6, and its close relative TRPV5 have low apparent affinity for PI(4,5)P_2_. The equivalent of the R575 residue in both TRPV6 and TRPV5 is G. Here we tested whether the lack of a positively charged residue at this position in TRPV5 and TRPV6 is responsible for the weak interactions of these channels with PI(4,5)P_2_.

We provide multiple lines of evidence that the G488R mutant of TRPV6 has higher apparent affinity for PI(4,5)P_2_ than the wild-type channel. 1. We found that current activity ran down much slower in the G488R mutant than in wild-type channels in excised inside-out patches. 2. Current levels after excision reflect the activity exerted by physiological resting PI(4,5)P_2_ levels, which is thought to be ~1% of the plasma membrane phospholipids^32^. The wild-type TRPV6 was activated upon long exposure to natural AASt PI(4,5)P_2_ more than 10-fold compared to current levels after excision, which reflect low apparent affinity for the lipid. The G488R mutant was activated to only ~2-fold that again is compatible with higher affinity. 3. The diC_8_ PI(4,5)P_2_ dose-response of the mutant channel was shifted to the left. 4. The mutant channel was inhibited much less than the wild-type upon depletion of PI(4,5)P_2_ by either high concentrations of wortmannin that inhibits PI4K, or by the voltage-sensitive 5-phosphatase ciVSP. The equivalent mutant G481R in TRPV5 was also not inhibited by ciVSP, indicating that the relatively low apparent affinity of TRPV5 is also caused by the lack of this positively charged residue.

Overall our data, together with earlier findings on TRPV1^21^, strongly support the role of the TM4-TM5 loop in interactions with PI(4,5)P_2_ in TRPV channels. These data are also analogous to data obtained in voltage gated K^+^ channels, where positively charged residues in this segment were proposed to interact with PI(4,5)P_2_ for Kv1.2 channels^48^ and for Kv7.1 channels^49^. The TM4-TM5 loop is unlikely to be the sole PI(4,5)P_2_ interacting region. In TRPV1 R575 and R579 in this region together with K694 in the proximal C-terminus were proposed to form the PI(4,5)P_2_ binding site, and mutations in these residues were shown to functionally affect PI(4,5)P_2_ sensitivity^21^. In addition, computational modeling also implied K571, Q561 in the TM4-TM5 loop and K688 in the proximal C-terminus of TRPV1 in PI(4,5)P_2_ binding, but the effects of mutating these residues were not confirmed experimentally^21^. In TRPV5, neutralizing mutation of the proximal C-terminal R599 residue (R599Q) reduced apparent affinity for PI(4,5)P_2_^14^. It will require further studies to delineate contribution of other residues and channel regions to PI(4,5)P_2_ interactions in TRPV6.

The G488R mutation also altered the specificity of activation by phosphoinositides, a phenomenon that was recently proposed to be an indication that a residue directly interacts with PI(4,5)P_2_^15^. Consistent with our earlier results, the wild-type TRPV6 channel was essentially not activated by 50 μM diC_8_ PI(4)P, and only to a small extent by higher concentrations. The concentration functionally equivalent to resting cellular levels was estimated to be 20 μM for diC_8_ PI(4)P, and 40 μM for diC_8_ PI(4,5)P_2_^22^. Thus endogenous PI(4)P is unlikely to significantly contribute to maintaining channel activity of the wild-type TRPV6. Accordingly, AASt PI(4)P had only a minimal effect on the wild-type TRPV6, and the 5′-phosphatase ciVSP robustly inhibited the channel. The G488R mutant showed much higher levels of activation by diC_8_ PI(4)P than the wild-type TRPV6 at any given concentration, and the mutant was also clearly activated by AASt PI(4)P. Based on our data, however, it is difficult to tell if the increased activation by PI(4)P is a true change in specificity, or part of a generalized increase in affinity for phosphoinositides.

The 5′-phosphatase ciVSP does not inhibit the G488R mutant of TRPV6. Is the lack of effect of ciVSP due to high affinity of this mutant for PI(4,5)P_2_, or because PI(4)P can also support channel activity? Based on our excised patch measurements (Fig. 2B,D), endogenous PI(4,5)P_2_ does not saturate G488R, as exogenous AASt PI(4,5)P_2_ still activates the channel above current levels after excision. Thus increased affinity for PI(4,5)P_2_ is unlikely to be solely responsible for the lack of inhibition by ciVSP and activation by PI(4)P is likely to also contribute to lack of inhibition by ciVSP. PI(4)P however was still less active than PI(4,5)P_2_, thus conversion of the latter to the former by the 5′-phosphatase is expected to at least partially inhibit the channel without increased affinity for PI(4,5)P_2_. We conclude that the lack of inhibition by ciVSP is likely caused by the combination of reduced specificity and increased affinity for PI(4,5)P_2_. Accordingly, inhibition of PI4K by wortmannin, which is expected to deplete both PI(4)P and PI(4,5)P_2_, moderately, but significantly inhibited the G488R mutant, even though to a lesser extent than the wild-type.

To have a better molecular understanding of how TRPV6 interacts with PI(4,5)P_2_, we have built a homology model based on the recently determined structure of the related TRPV1^41^, and docked PI(4,5)P_2_ to both the model of TRPV6 and the TRPV1 structure. We found that the binding energy predicted from our model was higher for TRPV1 than for TRPV6, which is consistent with experimental results showing higher affinity of TRPV1 for PI(4,5)P_2_. We also docked PI(4,5)P_2_ to the G488R mutant of TRPV6 and our model showed that PI(4,5)P_2_ interacted with the introduced positively charged residue. The mutant also showed higher binding energy for PI(4,5)P_2_ consistent with its higher apparent affinity for PI(4,5)P_2_ we found experimentally. The coincidence of TRPV1 modeling and experiments^21^, our modeling and experiments on TRPV6, and the quite clear parallel with Kir and Kv channels makes a strong point that the TM4-TM5 loop plays an important role in interactions with PI(4,5)P_2_ in TRPV channels.

TRPV6 is constitutively active, but it undergoes Ca^2+^-induced inactivation^50^. Both calmodulin (CaM)^33^,^35^,^51^ and Ca^2+^-induced activation of PLC and the resulting depletion of PI(4,5)P_2_^5^,^34^ have been shown to play a role in this phenomenon. Our data with ciVSP show that the mutant channel is essentially resistant to depletion of PI(4,5)P_2_. In agreement with the role of PI(4,5)P_2_ depletion in Ca^2+^-induced inactivation, the G488R mutant showed no Ca^2+^-induced inactivation in our protocol either in patch clamp or Ca^2+^ imaging experiments. The W695A/R699E mutant that eliminates CaM binding and inhibition in excised patches only showed a partial reduction in Ca^2+^-induced inactivation in the same patch clamp protocol we used here^33^. This suggests that PI(4,5)P_2_ depletion may be more important on this time scale in Ca^2+^-induced inactivation than CaM. It has to be kept in mind however that CaM and PI(4,5)P_2_ functionally compete^33^, thus increased affinity for PI(4,5)P_2_ may also decrease inhibition by CaM. Thus, our data do not exclude the contribution of CaM to inactivation.

In conclusion, to our knowledge, our data is the first showing that reintroducing a positive charge into a low PI(4,5)P_2_ affinity mammalian ion channel converts it to a channel with high affinity for this lipid. Our results, together with data in the literature, also establish an important role for the TM4-TM5 loop in PI(4,5)P_2_ activation of TRPV channels.

## Methods

### Xenopus oocyte preparation and RNA injection

Animal procedures were approved by the Institutional Animal Care and Use Committee of Rutgers New Jersey Medical School, and all animal procedures were performed in accordance with the approved guidelines. Oocytes were prepared from female *Xenopus laevis* frogs, as described earlier^52^. Briefly: oocytes were digested using 0.2 mg/ml collagenase (Sigma) in a solution containing 82.5 mM NaCl, 2 mM KCl, 1 mM MgCl_2_, and 5 mM HEPES, pH 7.4 (OR2) overnight for ~16 h at 18 °C in a temperature controlled incubator. Defolliculated oocytes were selected and kept at 18 °C in OR2 solution supplemented with 1% penicillin/streptomycin (Mediatech) and 0.1 mM CaCl_2_. This low concentration of Ca^2+^ was used to avoid Ca^2+^ overload and cell damage, which was especially prevalent in oocytes expressing the G488R mutant. cRNA was generated from the linearized human TRPV6 and TRPV6-G488R mutant in the pGEMSH oocyte vector, using the mMessage mMachine kit (Ambion). RNA, 5 ng for TEVC measurements, and 50 ng for excised patch experiments, was microinjected into each oocyte using a nanoliter injector system (World Precision Instruments). The experiments were performed 48–72 h after injection. The human TRPV6 was recently reported to have an alternate translation initiation site resulting in an extended N-terminus, resulting in increased trafficking to the plasma membrane^53^. The biophysical characteristics of the longer form did not differ from the originally described and well-characterized shorter version, which we used in this work.

### Excised inside-out patch clamp in Xenopus oocytes

Excised inside-out macropatch experiments were performed as described earlier^6^ using borosilicate glass pipettes (World Precision Instruments) of 0.8–1.2 megaohm resistance. After establishing giagohm resistance seals on devitellinized Xenopus oocytes, the currents were measured using an Axopath 200B amplifier (Molecular Devices) using a ramp protocol from −100 to +100 mV (0.25 mV/ms), immediately preceded by a 100-ms step to −100 mV. The protocol was applied every second; holding potential was 0 mV. The electrode pipette solution contained 96 mM LiCl, 1 mM EGTA, and 5 mM HEPES, pH 7.4 to allow monovalent inward currents. The bath solution contained 96 mM KCl, 5 mM EGTA, and 10 mM HEPES, pH adjusted to 7.4. The same solution was used to apply phosphoinositides locally to excised patches using a custom made gravity driven perfusion system. When plotting diC_8_ PI(4,5)P_2_ concentration response relationships, currents induced by the different concentrations were first normalized to the currents evoked in the same patch by 125 μM diC_8_ PI(4,5)P_2_ to reduce the effect of patch to patch variability in channel density. The individual points were fitted with a Hill equation using Origin 8, then for plotting in Fig. 2G, the data were renormalized for the maximum effect obtained from the Hill fits, both for wild-type TRPV6 and the G488 mutant.

### Two-electrode voltage clamp (TEVC) in Xenopus oocytes

TEVC measurements were performed as described earlier^54^; briefly oocytes were initially placed in a solution containing 97 mM NaCl, 2 mM KCl, 1 mM MgCl_2_, and 5 mM HEPES, pH 7.4, where TRPV6 currents are largely blocked by Mg^2+^ and trace amounts of Ca^2+^ in the medium. Monovalent currents were initiated with changing the solution to that identical to the pipette solution in excised patches (96 mM LiCl, 1 mM EGTA, and 5 mM HEPES, pH 7.4). Currents were recorded with thin-wall inner filament-containing glass pipettes (World Precision Instruments) filled with 3 M KCl in 1% agarose. Currents were measured with the same ramp protocol as described earlier for excised patch measurements.

### Mammalian cell culture and transfection

HEK293 cells were maintained in minimal essential medium with 10% fetal bovine serum (HyClone) and 1% penicillin/streptomycin (Mediatech) in a humidified, 5% CO_2_ incubator at 37 °C. Cells were transfected using the Effectene transfection reagent (Qiagen) with the following constructs: either the wild-type or mutant TRPV6 in pCMV-tag3A vector^55^, ciVSP in an EGFP-IRES vector, and pEGFPN1 as a transfection marker in experiments without ciVSP. The rat TRPV5 clone (CaT2)^56^ was sublconed into the pCDNA3.1(−) vector using standard molecular biology tools. The G481R mutation was introduced using the Quikchange mutagenesis kit (Agilent). Measurements were performed 36–72 h after transfection.

### Mammalian cell electrophysiology

Whole-cell patch clamp experiments were performed as described earlier^33^ on HEK293 cells in an extracellular solution containing 137 mM NaCl, 5 mM KCl, 10 mM glucose, 10 mM HEPES, with the pH adjusted to 7.4, to which 1 mM MgCl_2_, 2 mM CaCl_2_, or 2 mM EGTA was added, depending on the experimental conditions. Borosilicate glass pipettes (Sutter Instruments) of 2–4-megaohm resistance were filled with a solution containing 135 mM potassium-gluconate, 5 mM KCl, 5 mM EGTA, 1 mM MgCl_2_, 2 mM Na_2_ATP, 10 mM HEPES (pH 7.2). The cells were kept in extracellular solution containing 1 mM Mg^2+^ but no Ca^2+^ for 20 min before measurements. After formation of gigaohm resistance seals, whole-cell configuration was established, and currents were measured using an Axopatch 200B amplifier (Molecular Devices). Monovalent currents were initiated by switching to a solution without Mg^2+^ and 1 mM EGTA. Ca^2+^-induced inactivation was initiated using a solution containing 2 mM Ca^2+^ and no Mg^2+^, as describe earlier^5^,^33^. The data were collected and analyzed with the pCLAMP 9.0 software (Molecular Devices). Measurements were performed without capacitance compensation, but capacitative currents after voltage steps are not shown in the figures. All of the measurements were performed at room temperature (20–25 °C).

### Ca^2+^ imaging

Ca^2+^ imaging was performed as described earlier^34^ with some modifications. Briefly, HEK cells transfected with wild-type TRPV6 or the G488R mutant, and YFP as transfection marker. Cells were loaded with 1 μM Fura-2-lowAff AM (Abcam), a low affinity Ca^2+^-sensitive dye, for 30 min at room temperature in nominally Ca^2+^ free extracellular solution, supplemented with 0.1 mg/ml Bovine Serum Albumin. Fura-2-lowAff is the same as Fura-FF sold by other vendors, which was used in our earlier publication^12^. When loading was complete, the cells were washed with Ca^2+^ and Mg^2+^ free extracellular solution, and placed on the stage of an Olympus IX51 inverted microscope. Excitation light, alternating between 340 and 380 nm, was provided by a DeltaRAM monochromator-based light source from Photon Technology International (PTI). Data were collected with a Roper Cool-Snap camera, and analyzed with the ImageMaster V software (PTI). Ca^2+^ influx was initiated by a solution containing 2 mM Ca^2+^, and it was terminated by perfusing the cells with a Ca^2+^ free solution supplemented with 2 mM EGTA.

### Homology modeling

The TRPV6 atomistic model was built using the comparative homology modeling approach. The structure of apo TRPV1 (PDB code 3J5P)^41^,^42^ was utilized as a structural template. We extracted the pairwise sequence alignment between TRPV1 and TRPV6 from the multiple sequence alignment containing about 3,000 sequences published in ref. 57 and we manually curated it so that the insertions and deletions were always located outside the secondary structure elements. Then we generated 288 atomistic models of TRPV6 using ROSETTA^37^; among them, we selected the 20 with the highest scores. The G488R mutant of TRPV6 (20 structures) was generated using the Mutator plugin of VMD. The structure of the TRPV1 closed state (PDB code 3J5P) has missing regions, which were not resolved (the TM2-TM3 loop and the loop between TM5 and the pore helix). These parts were generated using the *ab initio* loop modeling of ROSETTA. Ten structures with the highest scores were selected for further docking simulations.

### Docking

To investigate the binding mode of PI(4,5)P_2_ we considered a model ligand and performed a series of stochastic optimizations of its positions with respect to the TRPV6 structure using the program AUTODOCK^36^. The model ligand is a simplified version of PI(4,5)P_2_ that contains the entire head group, the glycerol moiety and part of the acyl chains (up to the third carbon atom). This choice allowed us to explore thoroughly the conformational space by ignoring the large number of degrees of freedom describing the configuration of the flexible acyl chain. AUTODOCK TOOLS 1.5.6^36^ was used to assign the atom types and charges of PI(4,5)P_2_, TRPV6 and TRPV1. Since not all the binding poses of the model ligand correspond to a geometrically viable conformation of PI(4,5)P_2_, we post-processed the output of AUTODOCK and selected only those binding poses in which the acyl chain is correctly position with respect to the lipid bilayer. The conformational search was restricted to a specific region of the channel encompassing the TM4-TM5 linker, the N-term sections of TM1, TM3 and TM5, the C-term section and TM2 and TM4, the TM2-TM3 loop, the N-terminal section of the TRP box and the adjacent part of the cytoplasmic domain. The region was large enough to contain at least two adjacent subunits to allow for the identification of potential interactions at their interface. For each structure of TRPV6 (40 in total, 20 for the native channel, and 20 for the mutant) and TRPV1 (10 in total), 1,000 docking experiments were performed using the Lamarckian genetic algorithm allowing for a maximum of 250,000 energy evaluations.

### Data analysis

Data are represented as mean ± S.E.; statistical significance is calculated either with *t*-test or analysis of variance, as appropriate; *p < 0.05; **p < 0.01; ***p < 0.001

## Additional Information

**How to cite this article**: Velisetty, P. *et al*. A molecular determinant of phosphoinositide affinity in mammalian TRPV channels. *Sci. Rep.* **6**, 27652; doi: 10.1038/srep27652 (2016).

## Supplementary Material

Supplementary Information
