# Supplementary material for: A molecular determinant of phosphoinositide affinity in mammalian TRPV channels
Source: Sci Rep. 2016 Jun 13;6:27652. doi: 10.1038/srep27652 (PMC4904367; doi:10.1038/srep27652)
Supplement: Supplementary Information [file srep27652-s1.pdf]

Supplemental Material

A molecular determinant of phosphoinositide affinity in mammalian TRPV channels

Phanindra Velisetty[1], Istvan Borbiro[1], Marina A. Kasimova[2], Luyu Liu[1], Doreen Badheka[1], Vincenzo Carnevale[2], Tibor Rohacs[1]

[1]Department of Pharmacology, Physiology and Neuroscience, Rutgers – New Jersey Medical School, Newark, NJ 07103, [2]Institute for Computational Molecular Science at Temple University in Philadelphia, PA, 19122

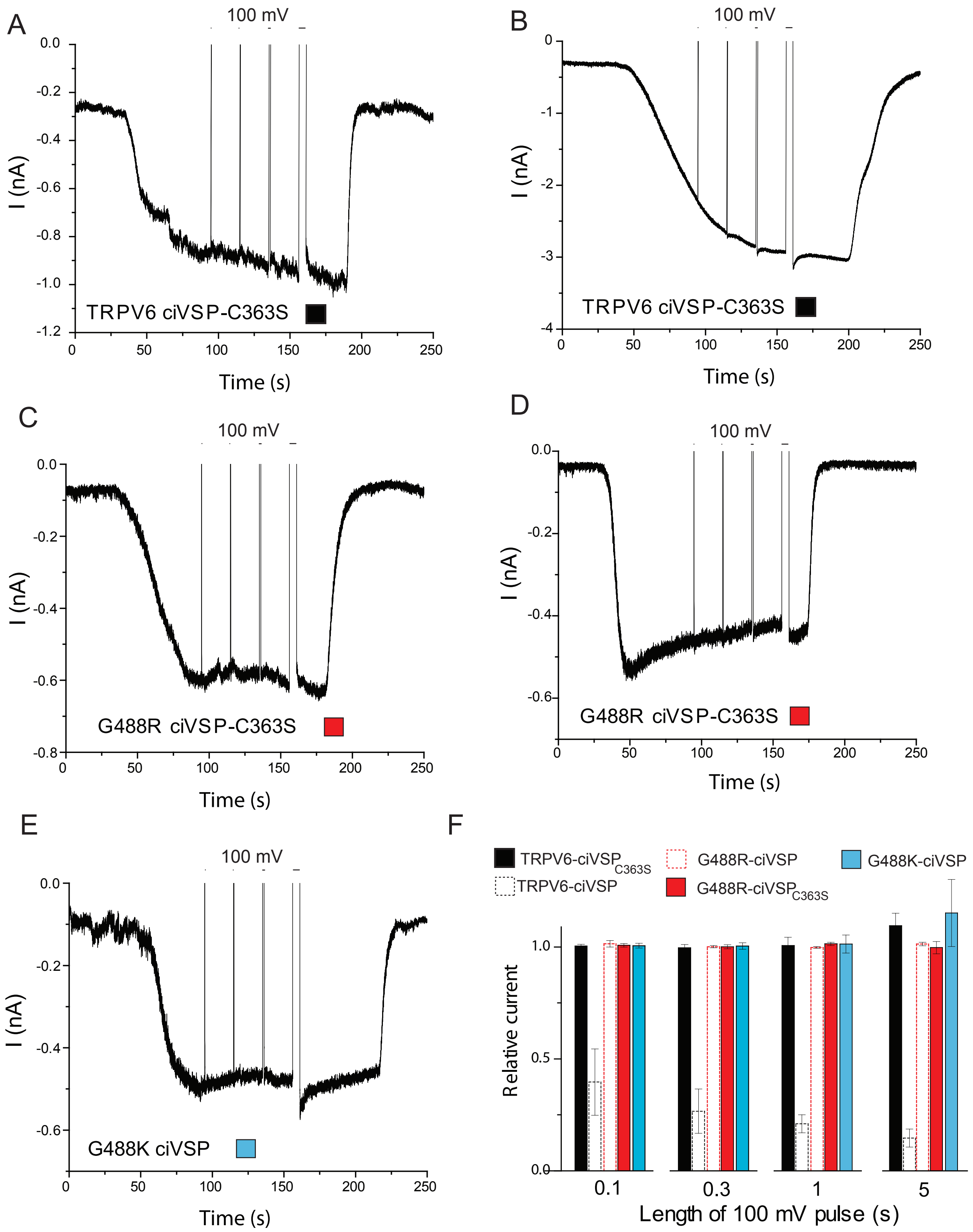

Figure S1. Lack of inhibition of wild-type and G488R TRPV6 by the phosphatase inactive ciVSP C363S mutant, and lack of inhibition of G488K TRPV6 by ciVSP. A-E Representative traces for measurements in HEK cells expressing active ciVSP or the inactive C363S mutant and various TRPV6 mutants, in an experimental setting identical to those in Fig. 3 and Fig. 4. *F* Summary data n=4-5, data for TRPV6 (dashed lines) and G488R ciVSP (dashed red lines) we re-plotted from Figure 3, with one additional experiment performed with wild-type TRPV6 as a positive control at the same time as the other experiments.

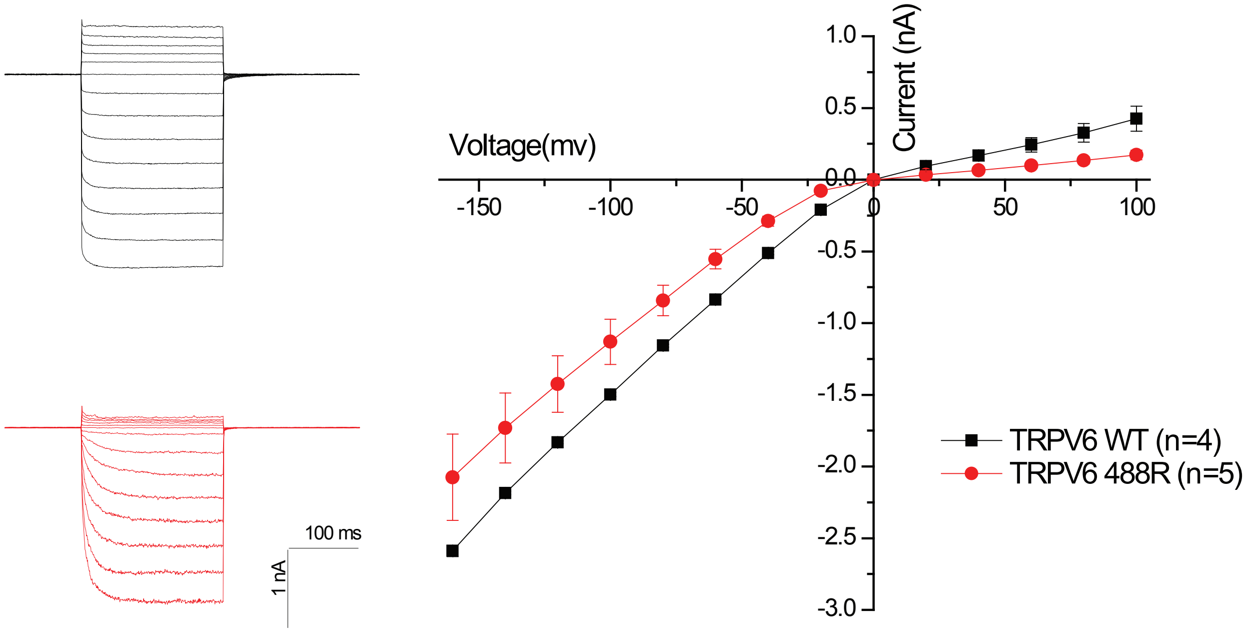

Figure S2. Similar inward rectification of monovalent currents via TRPV6 and TRPV6 G488R. Holding potential was -60 mV, voltage steps from -150 to 100 mV with 20 mV increments were performed.

**Experimental setting**	**Membrane potential**	**TRPV6**	**TRPV6-G488R**
HEK cell constant holding potential	-60 mV	-1.15 ± 0.19 nA (n=14)	-0.66 ± 0.12 nA (n=16, p=0.038)
HEK cell voltage steps	-60 mV	-0.837 ± 0.006 nA (n=4)	-0.553 ± 0.069 nA (n=5)
HEK cell voltage steps	-100 mV	-1.497 ± 0.015 nA (n=4)	-1.13 ± 0.157 nA (n=5)
Xenopus oocyte TEVC Ramp	-100 mV	-7.85 ± 1.04 µA (n=13)	-9.43 ± 1.15 µA (n=14)

Supplemental table. Summary of current amplitudes in different experimental settings.
